# Dual role of fucosidase in cancers and its clinical potential

**DOI:** 10.7150/jca.75840

**Published:** 2022-08-15

**Authors:** Jinxing Fu, Qing Guo, Yuan Feng, Peng Cheng, Anhua Wu

**Affiliations:** Department of Neurosurgery, The First Hospital of China Medical University, Shenyang, China

**Keywords:** Fucosidase, defucosylation, cancer, microenvironment, signaling pathway

## Abstract

Glycosidases and glycosyltransferases greatly impact malignant phenotype of tumors though genetics and epigenetics mechanisms. As the member of glycoside hydrolase (GH) families 29A, α-L-fucosidases (AFUs) are involved in the hydrolysis of terminal L-fucose residues linked via α-1,2, α-1,3, α-1,4 or α-1,6 to the reducing end of N-acetyl glucosamine (GlcNAc) of oligosaccharide chains. The defucosylation process mediated by AFUs contributes to the development of various diseases, such as chronic inflammatory diseases, immune disorders, and autoimmune diseases by reducing the interaction between fucosylated adhesion molecules supporting leukocyte extravasation. AFUs also impair crucial cell-extracellular matrix (ECM) interactions and presumably subsequent cell signaling pathways, which lead to changes in tumor function and behavior. There are two isoforms of AFUs in human, namely α-L-fucosidase 1 (FUCA1) and α-L-fucosidase 2 (FUCA2), respectively. FUCA1 is a p53 target gene and can hydrolyze different fucosylation sites on epidermal growth factor receptor (EGFR), thereby determining the activation of EGFR. FUCA2 mediates the adhesion between Helicobacter pylori and gastric mucosa and is upregulated in 24 tumor types. Besides, based on the participation of AFU in signaling pathways and tumor progression, we discuss the prospect of AFU as a therapeutic target.

## Introduction

**Figure 1:**
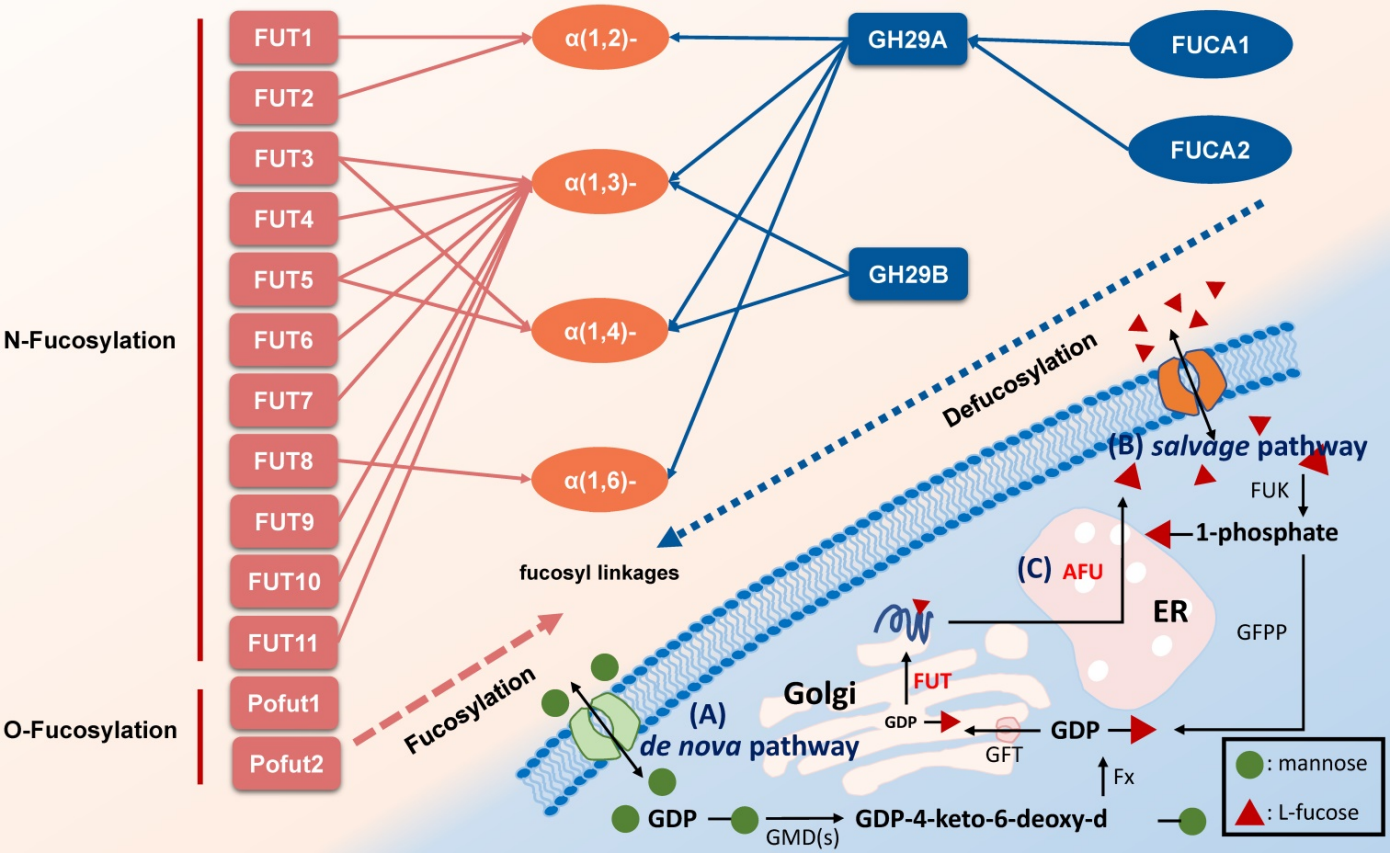
Pathways associated with fucose metabolism. **A.** The de nova pathway is the one of fucosylation pathway. GDP-mannose is converted to GDP-4-keto-6-deoxy-d-mannose by GMD(s). Then, under the catalyzation of Fx protein, it converted to GDP-L-fucose, followed by transfer to Golgi apparatus by GFT. In Golgi apparatus, the substrates including polysaccharide and protein are linked to GDP-L-fucose with the participation of FUT to form a fucosylated substrate, which is further transported to cell membrane 2, 10. **B.** The salvage pathway utilizes fucose transported into the cytosol from an extracellular origin or released by catabolism of fucosylated glycans in lysosome, and then transported into the cytosol. Fucose is transported across the plasma membrane through a poorly characterized mechanism, L-fucose-specific facilitated diffusion 7. The salvage pathway is enabled by FUK and GFPP, with L-fucose-1-phosphate as the metabolic intermediate, and the following steps are the same as the de novo synthesis pathway. **C.** The defucosylation pathway is a defucosylation process mediated by AFU in lysosome. GMD(s): GDP-mannose 4,6-dehydratase. Fx: Fx protein. GFT: GDP-L-fucose transporter. FUT: fucosyltransferase. GFPP: GDP-fucose pyrophosphorylase. FUK: L-fucokinase (L-fucose kinase). AFU: fucosidase

Glycosylation modification of polysaccharides on cell surface is closely associated with cancer initiation and progression. As a unique monosaccharide residue in glycans, fucose locates in the terminal position of oligosaccharides on human or other cells 1, 2. Fucose-containing polysaccharides mediate events such as blood transfusion reactions 3 and selectin-mediated leukocyte-endothelial adhesion 4. In mammals, L-fucose (6-deoxy-L-galactose) is incorporated into N-glycans, O-glycans and glycolipids by 13 fucosyltransferases (FUTs), all of which utilize GDP-fucose to modify target substrates 2. Among the FUTs discovered, FUT1-11 locate in the Golgi apparatus modify N-linked glycans via α-1,2, α-1,3, α-1,4 or α-1,6 link, however Pofut1 (FUT12) and Pofut2 (FUT13) often locate in the endoplasmic reticulum and act as O-fucosyltransferases that add fucose directly to polypeptides 5. Conversely, AFUs are lysosomal exoglycosidases catalyzing the hydrolysis of terminal L-fucose residues linked to the reducing end of GlcNAc of oligosaccharide chains 6, 7. Fucosylation and defucosylation are two key processes of fucoses metabolism (Figure 1).

To date, there are many studies and reviews on the function of FUTs. Most studies have shown the function of fucosylation and FUTs in the tumorigenesis and malignant progression such as tumor invasion and angiogenesis 8. The activity of FUTs is particularly high in the serum from highly malignant or metastatic tumor patients, such as colon cancer, breast cancer and liver cancer 9. Even within different subtypes of the same tumor such as melanoma, FUTs' expression varies 10.

Here, to improve the understanding of the functions of AFUs systematically, we reviewed the current reports of AFUs and defucosylation process in multiple cancers. We also summarized and discussed potential therapeutic strategies relevant to the fucosidase, which might help the development of novel clinic treatment strategies against cancers.

## The Structural Characteristics and Function of AFUs

In human, there are two isoforms of AFUs, namely FUCA1 and FUCA2 that are predominantly localized in the tissues such as liver 11, as well as plasma and fibroblasts 12, respectively. FUCA1 is encoded by a gene on chromosome 1p (1p34) 13 and FUCA2 is encoded by a gene linked to plasminogen on chromosome 6 (6q24) 14. They are both members of GH families 29A 15. To better understand the structure and differences of FUCA1 and FUCA2, we summarized amino acid modifications and possible key sites for catalysis using the AlphaFold and UniProt database 16 (Table 1). AFUs contain a catalytic N-terminal domain with a (β/α)8-TIM barrel structure and a C-terminal domain that needs to be further examined 6. Due to the existence of genetic polymorphism, AFUs have a certain degree of inter- and intra-tissue structural heterogeneity 17. Interestingly, a glutamic acid mutation to alanine of AFU produced by *lactobacillus casei* at positions 274 will switch AFU function from fucosidases to transfucosidases, which can transfer a fucose moiety to an acceptor such as GlcNAc from a simple fucosyl donor. This is the result of shifting equilibrium between open and closed conformations of an active-site loop 18. The conclusions have implications for us to understand the mutation of AFUs in tumors.

**Figure 2:**
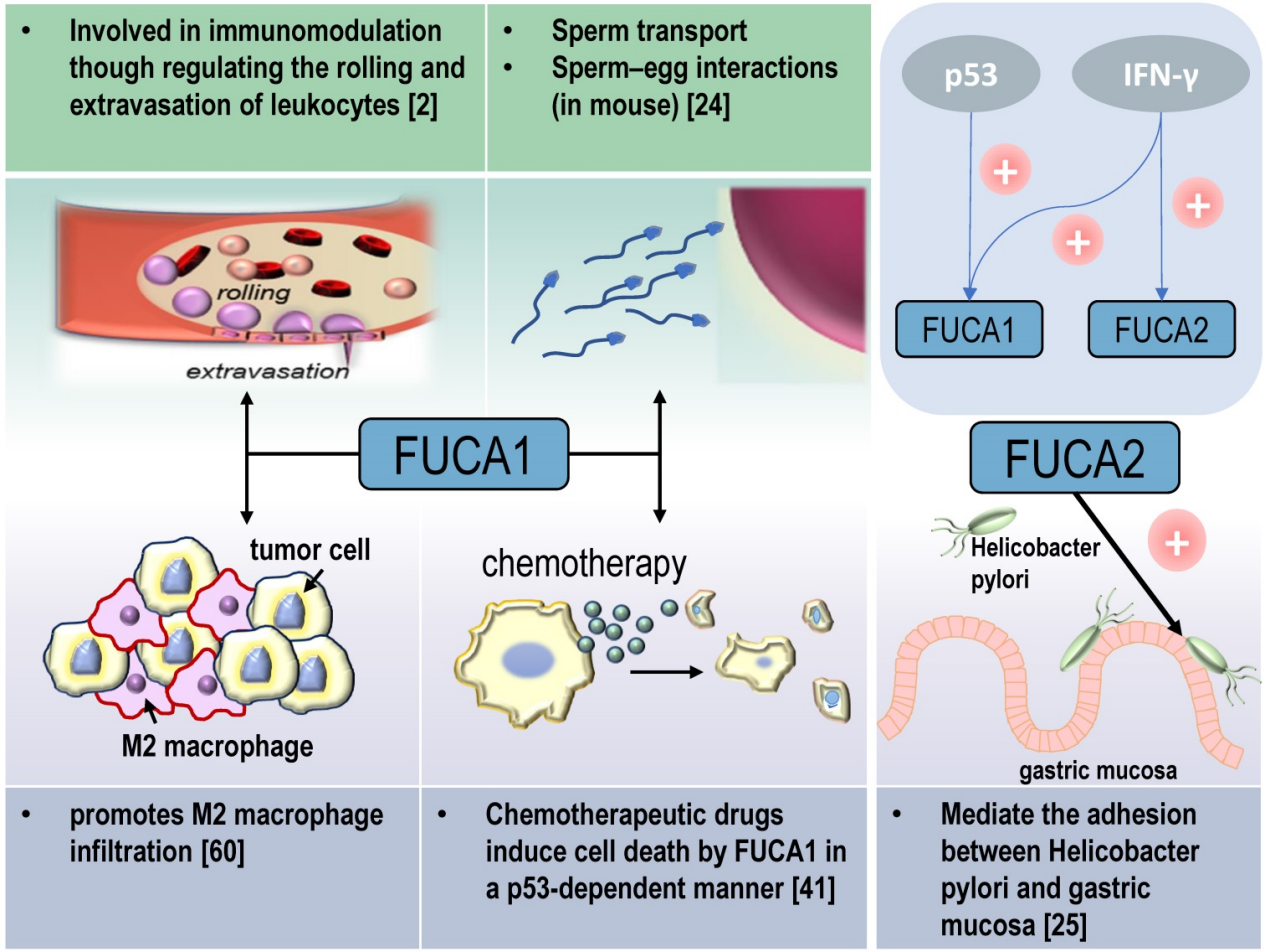
Physiological and pathological functions of AFU-mediated defucosylation. FUCA1 is involved in diversity physiological and pathological processes *in vivo*. FUCA2 mainly mediates the adhesion of Helicobacter pylori, especially to specific strains of gastric cancer and duodenal ulcer 94.

AFUs are main regulators of defucosylation in human metabolism. As glycoside hydrolases modifying the structure of sugar chains, FUCA1 and FUCA2 have wide substrate specificity for hydrolyzing α-1,2, α-1,3, α-1,4 or α-1,6-fucosyl linkages in glycolipids, glycoproteins and mucopolysaccharides 15, 18. Mammalian AFUs show greater activity on fucose linked α-1,2 to galactose compared to fucose linked α-1,3, α-1,4 or α-1,6 to N-acetylglucosamine 19. AFUs locate in the lysosome, and exhibit maximal activity at pH values between 4 and 7 19. In almost all mammalian tissues, the majority of the AFUs activity is in the soluble fraction except the human brain 20, 21. 10-20% of total cellular AFUs activity is detected in hematopoietic, epithelial and mesenchymal cells 21. In the human gut, *lactobacillus casei* can produce an AFU and involve in the catabolism of the core-fucosylated structures of mammalian N-glycoproteins 22. AFUs are involved in immunomodulation though regulating the rolling and extravasation of leukocytes 2. AFUs treatment could efficiently decrease the migration capability of monocytes without affecting cell viability and morphology. The degradation of fucose-containing epitope may be the key to transendothelial migration 23. FUCA1 can also participate in sperm transport and sperm-egg interactions 24. And FUCA2 has been reported to mediate the adhesion between Helicobacter pylori and gastric mucosa, especially for specific strains of gastric cancer and duodenal ulcer 25 (Figure 2).

## Dual Contribution of AFUs to Tumors

In cancer biology, cancer aberrant glycosylation reflected cancer-specific modification in glycan metabolism pathways. AFUs not only contribute to multiple malignant tumor development 26, 27, but also are involved in regulating pathological processes including immune evasion, invasion and metastasis of cancers 26, 28, 29. As the lysosomal enzyme, AFUs maintain the dynamic balance of fucose metabolism, and have been recognized to reflect and contribute to regulating the malignant behaviors of tumors **(**Table 2**)**. The activity change of AFUs in serum or tissues may be employed as an indicator of tumor burden, metastasis and response to anti-cancer treatment 30.

Mutations of FUCA1 can be found in various cancers, such as breast cancer, lung cancer and central nervous system cancer 31. Published research have shown FUCA1 elevation is detected in endometrial 32, thyroid 33, cervical cancer 34, hepatocellular carcinoma (HCC) 35, oral cancer and oral precancerous lesions 36, as well as gastric cancer 33 and glioblastoma (GBM) 26. Whereas a reduced expression and activity of AFU is observed in breast 37 and colorectal cancer 28. As for FUCA2, Zhong etc. has shown it is upregulated in most tumors such as thyroid carcinoma, lung squamous cell carcinoma, HCC, low grade glioma (LGG) and GBM and is significantly associated with poor survival 38. Due to the diverse metabolic characteristics and related molecular mechanisms, AFUs may play even completely opposite roles in different types of cancer.

### Signaling molecules that interact with FUCA1 involving in the progression of tumors

#### p53: Transcription Factor of FUCA1

Tumor suppressor p53 is a key regulator of programmed cell death 39. p53 modulates multiple cellular pathways associated with tumor suppression, and p53-induced programmed cell death is also an important factor in the response to chemotherapy 40 (Figure 2). *FUCA1* is a p53 target gene 31. p53 transcriptionally activates FUCA1 27 and regulates fucosidase activity via FUCA1 up-regulation. Chemotherapeutic drugs induce cell death by FUCA1 and fucosidase activity in a p53-dependent manner 41. Research by Ezawa et al. showed FUCA1 had tumor-suppressing activity. The expression of FUCA1 inducted apoptosis in COS7 cells and *FUCA1* knockdown enhanced the proliferation of H1648 cells 31.

#### EGFR: Dual Effect under Different Defucosylation Processes

**Figure 3:**
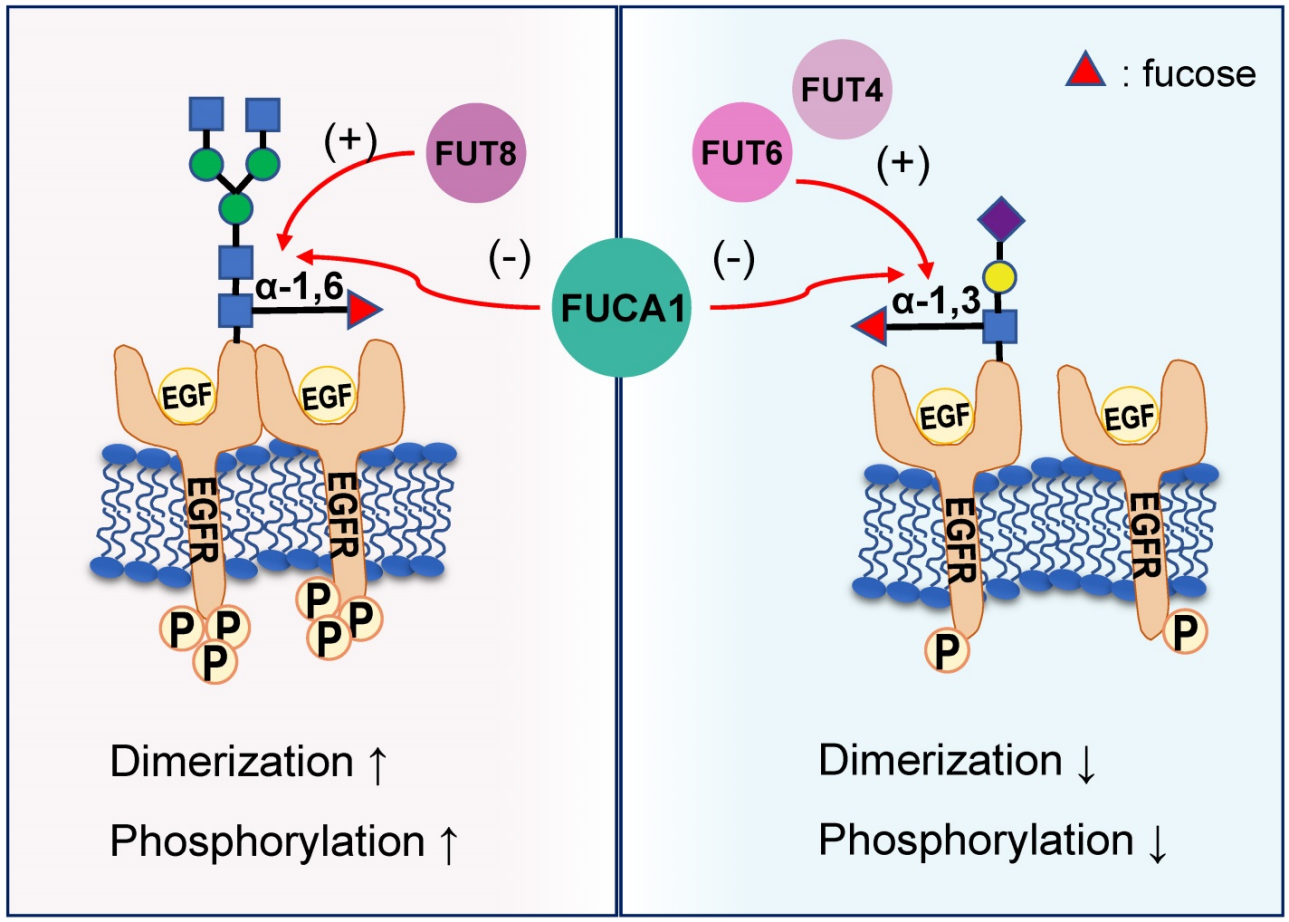
Different EGFR fucosylation sites lead to different EGFR activation. Core fucosylation of EGFR catalyzed by FUT8 contributes to the increased dimerization and phosphorylation of EGFR. However, the terminal α-1,3-fucosylation catalyzed by FUT4 or FUT6 would suppress EGFR dimerization and phosphorylation upon EGF treatment. FUCA1 can cleave the α-1,6 or α-1,3 fucosyl link on EGFR and produce the opposite result.

EGFR plays a critical role in the pathogenesis of many cancers 42. Seven related ligands active human EGFR and generate different signal outputs from the receptor 43. The dimerization of ligands is crucial for them to active the EGFR 44. Zhen, etc. has reported twelve N-linked glycosylation sites in the extracellular region of EGFR 45. Among them, core fucosylation of EGFR catalyzed by FUT8 contributes to the activation of EGFR 46, and the dimerization and phosphorylation of EGFR 47. However, the terminal α-1,3-fucosylation catalyzed by FUT4 or FUT6 would suppress EGFR dimerization and phosphorylation upon EGF treatment 47. FUCA1 can repress EGFR signaling by cleave the α-1,6 fucosyl link on EGFR, which has shown a direct link between the removal of fucosyl linkages and tumor suppression 31 (Figure 3). FUCA1 can also inhibit EGFR signal transduction and its downstream signaling by inhibiting Akt phosphorylation 31. Collectively, different fucosylation sites of EGFR will cause different modification results, which also lead to differences in results after AFUs treatment. Perhaps in the future, some AFUs targeting the core fucosylation site can be designed to specifically inhibit tumor progression caused by EGFR.

#### Integrin and Epithelial-mesenchymal transition (EMT): Targets of AFU to Inhibit Tumor Metastasis

The process of epithelial-to-mesenchymal transition (EMT) plays an important role in the invasion and metastasis of solid cancer cells 48. Decreased FUCA1 results in increased expression of fucosylated N-glycans in transforming growth factor-beta (TGF-β) -induced EMT in non-malignant bladder transitional epithelial HCV29 cells 49. Integrins are involved in cell-cell and cell-matrix interactions, which regulate processes such as intracellular signaling and cancer metastasis 50. The ECM-integrin signaling is involved in the regulation of cell migration 51. Considerable evidence indicates that glycosylation modulates the function of integrins 52, such as the ability of cells to adhere to fibronectin and laminin-1 53. The core fucosylation of α3β1 integrins stimulates embryonic fibroblasts migration via laminin-5, and loss of core fucosylation will result in the deficiency of α3β1 integrin function and the reduction of integrin-stimulated phosphorylation of focal adhesion kinase (FAK) 54, 55. In bladder cancer, calreticulin regulated cell adhesion through α-1,2-linked fucosylation of β1 integrin and a FUT1-specific fucosidase diminished the activation of β1 integrin 56. In breast cancer, treatment of the cells with AFU decreases the colocalization of fucose with β1 integrins 57, partially inhibited cell adhesion to laminin-α5 chain-derived peptides 58. The evidences suggest AFU can reverse the activation and function of integrins induced by fucosylation.

### FUCA1 promotes tumor progression by affecting the tumor microenvironment (TME)

#### Immune Cells

Tumor-associated macrophages (TAMs) are notorious for their roles in constructing immune suppressive TME and promoting tumor immune evasion. This contributes to the progression and metastasis of multiple types of cancers 59. AFUs have shown their potential in regulating macrophage behaviors. A recent study shows that FUCA1 is one of six M2 macrophage co-expressed genes related to M2 macrophage infiltration in renal clear cell carcinoma 60. FUCA1 promotes M2 macrophage infiltration and it serves as a biomarker for the phenotype and immune microenvironment of renal clear cell carcinoma 60 (Figure 2). In consistent with this, silencing *FUCA1* in GBM efficiently inhibits the infiltration of macrophages by downregulating the expression of chemokine C-C motif ligand (CCL)2/CCL5 26. α-L-fucose may comprise an essential part of the macrophage membrane receptor for lipopolysaccharide (LPS), and this may induce the resistance of FUCA1-treated tumor cells to macrophage-mediated cytotoxicity 61. Because of interference with the binding of T-cell fibronectin fucose residues to monocyte fucose receptors, T-cell treated by FUCA1 abolishes their fibronectin mediated agglutinating activity for human monocytes 62. Additionally, lysosome and lysosomal enzymes are involved in B cell apoptosis. AFUs increase activities in human tonsil B lymphocytes undergoing *in vitro* spontaneous apoptosis 63. The activity of AFUs is decreased in B cells of chronic lymphocytic leukaemia when compared with control or other leukaemic lymphoid cells 64.

#### ECM

Cancer cells draw support from stromal cells in TME to escape antitumor therapeutic strategies 65. This cancer-stromal cell interaction may dampen immunosurveillance and contribute to the immune suppressive mechanisms in TME. In melanoma, the fucose salvage related α-1,2 fucosylation pathway is involved in inhibiting invadopodia formation and ECM degradation 66. FUCA1 has been identified to effectively reduce the invasiveness of cancer cells in breast cancer through removing fucose-rich polysaccharides on cell surface and inhibiting fucosylation 37. Specifically, FUCA1 decreases the interaction of tumor cells with a wide variety of ECM components, including fibronectin, laminin, type I collagen, hyaluronic acid and other extracellular matrix, which weaken the adhesion between tumor cells and ECM 57. Additionally, FUCA1 could inhibit intercellular adhesion of breast cancer cells though down-regulating their CD44, CD15, and matrix metalloproteinases (MMP) -9 expression. The regulatory role of FUCA1 on two cancer stemness marker, CD44 and CD15, implies its potential function in recurrent of breast cancer 67. FUCA1 expression in breast cancer with lung metastasis lesion is lower than that of breast cancer without lung metastasis 29. *In vitro* experiments using AFU to remove α-L-fucose residues from the surface glycoprotein of breast cancer cells could decrease their adhesion-migration ability. AFU mediating defucosylation on these cells reduces their rolling ability and impairs the interaction between them and ECM, indicating the key role of FUCA1 in modulating tumor progression 57. Further studies indicated that FUCA1 could downregulate MMP-9 expression and activity, thereby diminishing the invasive ability of intrahepatic cholangiocarcinoma 68.

## Clinical significance of AFUs in the early diagnosis and prognosis predication of cancer

Previous studies demonstrate the change of AFUs in serum and tumor samples of cancer patients 28, 38. AFUs could be employed as an indicator of tumor burden, metastasis and response to anti-cancer treatment 30. This arouses the interest of researchers because the detection of expression and activity changes of AFUs in serum and/or tissues may help the early diagnosis of cancer patients.

Firstly, AFU is a useful serum marker for the diagnosis of HCC; however its diagnostic value to detect early-stage HCC has not yet been investigated comprehensively 69. But a recent development of an AFU immunoassay is said to further improve the sensitivity of early detection in HCC patients 70. Moreover, in ovarian cancer patients, the activity of AFU had a statistically significant deficiency 71 and could be used as a marker for advanced ovarian cancer and be unaffected by the histological type of ovarian cancer. Its sensitivity and specificity are similar to CA125, the well-known tumor marker in ovarian cancer 72. The preoperative serum AFU shows a significant association with the prognosis of HCC 73 and esophageal squamous cell carcinoma (ESCC) 74. Furthermore, AFU activity appears to be a good independent prognostic factor of tumoral recurrence in colorectal cancer (CRC) 75 and triple-negative breast cancer 29. If we combine the detection of serum AFU, alpha-fetoprotein (AFP) and thymidine kinase 1 in the diagnosis of HCC, the sensitivity will be significantly improved because they have complementary roles 76. In addition, the combination of AFU and CD26 has high sensitivity and specificity for the detection of early CRC 75. But the results weren't all as good. In pure urothelial carcinoma (UC), the retrospective study indicated that preoperative AFU levels cannot serve as a reliable predictor for malignant degree and differential diagnosis 77.

Moreover, recent study reveals that FUCA2 is also a diagnostic and prognostic biomarker a therapeutic target in pan-cancer 25. The expression of FUCA2 is associated with immunosuppressive microenvironment such as TAMs, and studies have indicated that FUCA2 is overexpressed in 24 tumor types 38. FUCA2 also can quantify AFU concentrations in human blood serum for early tumor detection such as HCC 70.

## Current development of defucosylation inhibitors and related anti-tumor treatment strategies

Glycobiology field is a target with therapy potential that is poorly explored. At present, some glycans are known to impair or modulate tumor treatment. For example, in immunotherapy, N-linked glycosylation of programmed cell death ligand 1 (PD-L1) maintains its protein stability and interacts with PD-1, which in turn promotes the evasion of T cell immunity. However, N-glycans can inhibit the recognition of PD-L1 antibody on the surface of tumor cells 78. Through database analysis, Zhong et al. found that the expression of FUCA2 was positively correlated with the immunosuppressive gene such as PD-L1 38. PD-L1 may be inhibited by targeting FUCA2. But there is no related research yet. Cell surface sugar engineering can effectively and specifically guide chimeric antigen receptor (CAR) T-cell into tissue sites that contain E-selectin expressing endothelial beds to locate the lesion tumor site more effectively 79. In glycosylation-mediated cancer-targeted drug delivery, fucose can facilitate the improvement of the target abilities of carriers 80. For the subset of tumors overexpressing AFU, targeting fucose with AFU could function as a drug delivery vehicle. For instance, pyrrolidine-ferrocene conjugates are composed of L-fuco-configured dihydroxypyrrolidine, which acts as an AFU ligand, is equipped with a cytotoxic ferrocenylamine moiety, and has a strong anti-tumor proliferation ability 81.

Deregulated glycosylation is a common post-transcriptional modification in cancers. Inhibition aberrant glycosylation or restoring normal glycosylation patterns have been explored to develop potential therapeutics for various types of cancers. According to previous reports, we summarize more detailed current available defucosylation inhibitors in Table 3. Most of the following reported AFU inhibitors are carried out from the perspective of molecular chemistry. If these inhibitors are to be used in the clinic, many animal experiments are needed to verify their effects and toxicity *in vivo*. Only then may AFU be used in clinic as a potential therapeutic target.

Among them, L-fuconojirimycin 82-84, deoxymannojirimycin (DMJ, 1,5-dideoxy-1,5-imino-D-mannitol) 85 and deoxyfuconojirimycin (DFJ, 1,5-dideoxy-1,5-imino-L-fucitol) 86 are effective AFU inhibitors. β-L-homofuconojirimycin (β-HFJ) is a powerful competitive inhibitor of human liver AFU with highly selective 87. N-(2-fluorophenyl)-2β-deoxyfuconojirimycin acetamide is a more efficient and selective AFU inhibitor, which display very potent and selective inhibition of AFU, and exhibits about 18-fold stronger effects on AFU than original DFJ 88. In addition, (-)-adenophorine 89 and all-cis pyrrolidine such as racemic aminofuranofucitol 90 has also been identified as an effective inhibitor of AFU. And aminomethyl pyrrolidine such as D-galacto aminomethyl pyrrolidine has also a AFU inhibitory activity 91. On human hormone-independent breast cancer cell line MDA-MB-231 and the human melanoma cell line SK-MEL28, ferrocenyl-iminosugar conjugate show significant anti-cancer activity 92.

## Discussion

Glycosylation modifications, the structural changes of carbohydrates on tumor cell surface, not only drive critical pathological processes in cancers like EMT, but also regulate their responses to therapeutic strategy. As the key enzymes in defucosylation, AFUs are not only involved in physiological processes such as cell recognition and immune response, but also are associated with tumor immunosuppression and malignant progression. And although current studies confirm the occurrence of aberrant fucose metabolism in a variety of cancer cells, there are several issues worth further investigations. Firstly, the detailed effects of fucosylated sugar chain change of fucose modified protein on tumorigenesis need further exploration. Secondly, it remains to be studied how fucosidase and fucosyltransferase affect the structure of polysaccharide chains, and which glycoproteins or glycolipids on cancer cell surfaces would be affected by these enzymes. The interactions and dependencies between them also deserve attention. Further investigation on the functions of AFUs including their mutant forms will help us better understand the defucosylation mechanism related to cancer initiation and progression. Mutations of FUCA1 can be found in various cancers and have also been linked to congenital disorders such as fucosidosis. Certain point mutants in FUCA1 gene could change its function from remove to add fucose residues to monoclonal antibody N-glycans, with significant impacts on their effector functions. This shows that the activity of the enzyme is controlled by distinct open and closed conformations of the active-site loop. Some mutations shift the balance to the open conformation and promote transfucosylation rather than hydrolysis 18. It will provide novel ideas for the development of fucosidase related cancer therapeutics. Interestingly, recent studies demonstrate that, similar to the glycosylation of PD-L1 in tumor cells, fucosylation of PD-1 could promote its stability and presentation on the surface of T cells 93. Mouse T cells with *FUT8* gene knockout or drug inhibition (such as 2-fluoro-L-fucose) decrease their core fucosylation and PD-1 expression. These cells demonstrate stronger cytotoxicity and more effective killing effect on melanoma and lung cancer cells 93. This implicates the clinical potential and possible synergetic effect of therapeutic strategies combined defucosylation regulation and immune checkpoint blockade like PD-1. Besides, although there are fewer studies on FUCA2 than on FUCA1, the existing researches are enough to explain the important value of FUCA2, including its diagnostic value for tumors and its role in the adhesion between Helicobacter pylori and gastric mucosa.

p53 is a tumor suppressor. p53 transcriptionally activates FUCA1, and chemotherapeutic drugs induce cell death by FUCA1 in a p53-dependent manner. In different tumors, the content and activity of AFUs vary widely. Due to the close relationship between p53 and FUCA1, it is worth considering whether the status of p53 in various tumors can affect the duality of FUCA1. Besides, the contradictory observations may reflect an inadequate host-feedback mechanism to regulate the 'fucose-burden' in serum and in tissue, respectively. It is precisely because of the difference and the existence of mutations of AFUs that it is far from enough to find a certain AFU inhibitor. It is necessary to analyze specific problems and explore its possible mechanism in depth. It is still necessary to further verify the effect and safety of AFU inhibitors *in vivo*.

## Figures and Tables

**Table 1:** Feature table of FUCA1 and FUCA2.

Gene	FUCA1	FUCA2
Protein	Tissue alpha-L-fucosidase	Plasma alpha-L-fucosidase
Localization	tissue	plasma
Subcellular location	Lysosome	
Length	466	467
Possible key site for catalysis	296	294
Human chromosome	1p34	6q24
Protein family	GH29A	
Mass (Da)	53,689	54,067
Amino acid modifications	170→Phosphothreonine 241→N-linked 268→N-linked 382→N-linked	171→N-linked 239→N-linked 301→Phosphoserine 377→N-linked
Structure	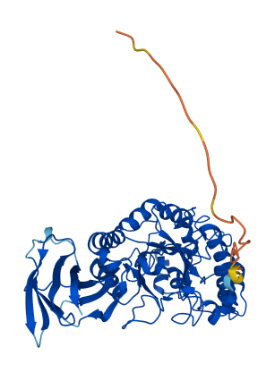	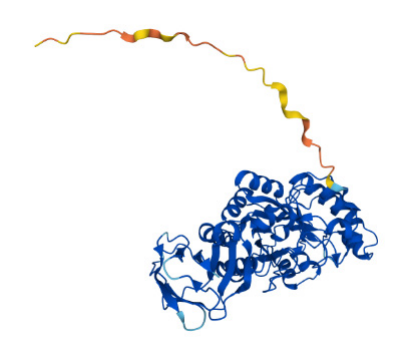

Representation of the structure in FUCA1 and FUCA2 is listed from AlphaFold database (https://www.alphafold.ebi.ac.uk/). AlphaFold produces a per-residue confidence score (pLDDT) between 0 and 100. Among them, blue represents the confidence of the model is very high (pLDDT>90), light blue represents credible (90>pLDDT>70), yellow represents low (70>pLDDT>50) and orange represents very low (pLDDT<50). Some regions with low pLDDT may be unstructured in isolation.

**Table 2:** AFUs' expression or activity in tumors.

Enzyme	Tumor types		Detailed
FUCA1	downregulated	Breast cancer 37	FUCA1 has been identified to effectively reduce the invasiveness of cancer cells in breast cancer 37. FUCA1 could inhibit intercellular adhesion of breast cancer cells 67.
		CRC 28, 31	Both of FUCA1 mRNA and protein in adenocarcinoma-derived tissue were significantly reduced compared with normal mucosa. This may be related to the aberrant methylation in the promoter region of FUCA1 28. FUCA1 could induce p53-mediated cell apoptosis in colon cancer, and p53 could directly regulate FUCA1 to promote cell death induced by chemotherapy 41.
		Neuroblastoma 95	*FUCA1* was recently shown to be down-regulated in neuroblastoma with unfavorable characteristics 95, 96.
	upregulated	Cervical cancer 34	Vesce F etc. showed the activity of AFU was increased in cervical cancer tissue than benign conditions 34.
		Endometrial cancer 32	Researchers have found an elevation of AFU activity in malignant endometrial tissues 32, 34.
		Gastric cancer 33, 97	Gastric cancer showed enhanced AFU activity compared to normal tissue^33^. Additionally, gastric cancer patients show an enhanced AFU activity in serum and tumor tissues compared with normal samples 33, 97.
		ESCC 98	High FUCA1 expression and high MMP-9 expression were potential predictors of shorter overall survival in ESCC 98.
		Glioma 26	An integrated analysis with TCGA and Chinese Glioma Genome Atlas (CGGA) confirmed the overexpression of FUCA1 in high-grade glioma, which is positively correlated with the poor survival. The inhibition of FUCA1 could efficiently inhibit the growth of glioma cell *in vivo* and* in vitro*. FUCA1 may become a potential target for the treatment of gliomas by promoting their autophagy 26.
		HCC 35, 99	Serum AFU activity was significantly higher in patients with HCC than in normal subjects 35, 99. AFU was a prognostic indicator for HCC 73.
		Oral cancer 36	Serum and salivary AFU activity were significantly higher in oral precancerous conditions and oral cancer patients compared to the controls 100, and were higher in patients with metastasis as compared to nonmetastatic patients 101. Serum AFU was identified as a useful marker for close monitoring of patients during post-treatment follow-up 36.
	uncertain	Ovarian cancer 34, 72	The investigation on female genital tract tumors revealed the lowest level of serum AFU activity in ovarian cancer patients, in comparison with benign ovarian tumors and the health female patients 72. And low levels of AFU indicated mildly increased risk for ovarian cancer 102. This might be related to the inhibition of AFU on the proliferation and colony formation capabilities of ovarian cancer cells 103. However, Vesce F, etc. showed the activity of AFU was increased in ovarian cancer tissue than benign conditions 34.
		Thyroid tumours 27, 33	FUCA1 RNA expression levels were found to be lower in poorly differentiated, metastatic and anaplastic thyroid cancer samples (ATCs), while they were higher in papillary thyroid cancer samples (PTCs) and in normal thyroid tissues. The down-regulation of FUCA1 is related to the increased aggressiveness of thyroid cancer 27, 33.
FUCA2	upregulated	24 tumor types 38	*Helicobacter pylori* is the primary cause of gastric cancer. A previous study identified that FUCA2 was essential for *H. pylori* adhesion, especially the gastric cancer-specific strains, and may help their defense strategy to escape host surveillance 25.

In some clinical studies of tumors, AFU, rather than specific FUCA1 or FUCA2, was used to measure the level of fucosidase. Although it is more convenient to use the level of serum fucosidase to detect in clinical practice, FUCA1 is more common in the current study. So we will summarize the part of the unclassified studies to the 'FUCA1' part for summary.

**Table 3:** The development of defucosylation inhibitors and related anti-tumor treatment strategies.

Inhibitors	Characteristic	Ki / IC_50_	Structural formula	Refs.
Ferrocenyl-iminosugar conjugate	A tight binding affinity for fucosidases and the fucosidase-targeting pyrrolidine could localize their deleterious effect	Ki=23 nM^†^	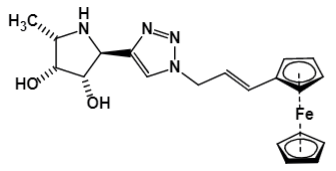	92
L-fuconojirimycin (5-amino-5-deoxy-L-fucose)	Inhibit FUCA1 by covalent binding of cyclophellitol aziridine to AFUs in a competitive activity-based protein profiling setting	Kiα=3.0 nM^†^	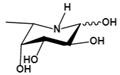	82-84
		Kiβ=1.0 nM^†^		
		Ki=1.7 nM^u^	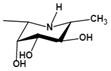	
Deoxyfuconojirimycin (DFJ) (1,5-dideoxy-1,5-imino-L-fucitol)	pH-dependent A powerful competitive inhibitor of AFU with highly specific	Ki=4.8* or 6.2 nM^ǂ^ Ki=10 nM^¶^	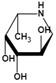	85, 87
*N*-methyl-DFJ	pH-dependent	Ki=50 nM^u^	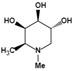	85
N-(2-fluorophenyl)-2β-deoxyfuconojirimycin acetamide	Change not only the inhibition potency but also the inhibition profile	IC_50_=0.012 μM^u^	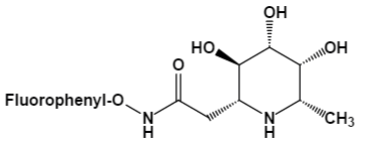	88
β-L-homofuconojirimycin (β-HFJ)	A powerful competitive inhibitor of human liver AFU with highly selective	Ki=5.3 nM^ǂ^ Ki=10 nM^§^	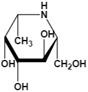	87
Inhibitors	Mechanism	Ki / IC_50_	Structural formula	Ref.
Deoxymannojirimycin (DMJ) (1,5-dideoxy-1,5-imino-D-mannitol)	A potent inhibitor of AFU	Ki=4.7 μM^ǂ^	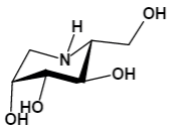	85, 87
*N*-methyl-DMJ (Me-DMJ)	Reduce or abolish inhibition towards bovine AFUs Enhance inhibitory potential towards the human AFUs	Ki=30 μM^ǂ^	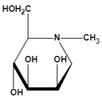	
Rha-DMJ (6-O-a-l-rhamnopyranosyl-DMJ)	A specific inhibitor of AFUs The first naturally occurring glycoside of DMJ Much stronger inhibition towards mammalian AFUs than DMJ	Ki=60 nM^ǂ^	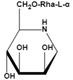	
(-)-adenophorine	a moderate and distinctive AFU inhibitor	IC_50_=72 μM^†^	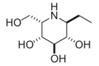	89
all-cis pyrrolidine	a potent specific inhibitor of AFU	Ki=15 nM^u^	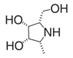	90
*D-galacto* aminomethyl pyrrolidine	a selective inhibitor	Ki=1.3 mM^u^	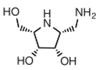	91

Ki: Inhibition constants. †: bovine kidney. ǂ: bovine epididymis. §: human placenta. ¶: human liver. u: unspecified

## Abbreviations

AFP: Alpha fetoprotein
AFUs: α-L-fucosidases
β-HFJ: β-L-homofuconojirimycin
CAR: chimeric antigen receptor
CRC: colorectal cancer
DFJ: Deoxyfuconojirimycin
DMJ: Deoxymannojirimycin
ECM: Extracellular matrix
EGF(R): Epidermal growth factor (receptor)
EMT: Epithelial-mesenchymal transition
ESCC: Esophageal squamous cell carcinoma
FAK: focal adhesion kinase
FUTs: Fucosyltransferases
FUCA1: α-L-fucosidase 1
FUCA2: α-L-fucosidase 2
GBM: Glioblastoma
GH: glycoside hydrolase
GlcNAc: N-acetyl glucosamine
HCC: Hepatocellular carcinoma
LGG: Low grade glioma
LPS: lipopolysaccharide
MMP: Matrix metalloproteinases
PD-(L)1: programmed cell death (ligand) 1
TAMs: Tumor-associated macrophages
TGF-β: Transforming growth factor-beta
TME: Tumor microenvironment
UC: Urothelial carcinoma

## References

[1] Werz D, Ranzinger R, Herget S, Adibekian A, von der Lieth C, Seeberger PJAcb (2007). Exploring the structural diversity of mammalian carbohydrates ("glycospace") by statistical databank analysis. ACS Chem Biol.

[2] Schneider M, Al-Shareffi E, Haltiwanger R (2017). Biological functions of fucose in mammals. Glycobiology.

[3] Lowe JJBsch (1993). The blood group-specific human glycosyltransferases. Bailliere's clinical haematology.

[4] Springer TJC (1994). Traffic signals for lymphocyte recirculation and leukocyte emigration: the multistep paradigm. Cell.

[5] Shan M, Yang D, Dou H, Zhang L (2019). Fucosylation in cancer biology and its clinical applications. Prog Mol Biol Transl Sci.

[6] Intra J, Perotti M, Pavesi G, Horner DJG (2007). Comparative and phylogenetic analysis of alpha-L-fucosidase genes. Gene.

[7] Wiese TJ, Dunlap JA, Yorek MA (1994). L-fucose is accumulated via a specific transport system in eukaryotic cells. Journal of Biological Chemistry.

[8] Pinho S, Reis CJNrC (2015). Glycosylation in cancer: mechanisms and clinical implications. Nat Rev Cancer.

[9] Sen U, Guha S, Chowdhury J (1983). Serum fucosyl transferase activity and serum fucose levels as diagnostic tools in malignancy. Acta medica Okayama.

[10] Keeley TS, Yang S, Lau E (2019). The Diverse Contributions of Fucose Linkages in Cancer. Cancers.

[11] Van Hoof F, Hers H (1968). Mucopolysaccharidosis by absence of alpha-fucosidase. Lancet.

[12] Van Elsen A, Leroy J, Wauters J, Willems P, Buytaert C, Verheyen K (1983). In vitro expression of alpha-L-fucosidase activity polymorphism observed in plasma. Human genetics.

[13] Goss S, Harris H (1977). Gene transfer by means of cell fusion. II. The mapping of 8 loci on human chromosome 1 by statistical analysis of gene assortment in somatic cell hybrids. Journal of cell science.

[14] Eiberg H, Mohr J, Nielsen L (1984). Linkage of plasma alpha-L-fucosidase (FUCA2) and the plasminogen (PLG) system. Clinical genetics.

[15] Sakurama H, Tsutsumi E, Ashida H, Katayama T, Yamamoto K, Kumagai H (2012). Differences in the substrate specificities and active-site structures of two α-L-fucosidases (glycoside hydrolase family 29) from Bacteroides thetaiotaomicron. Bioscience, biotechnology, and biochemistry.

[16] UniProt Consortium (2021). UniProt: the universal protein knowledgebase in 2021. Nucleic Acids Res.

[17] Kretz K, Cripe D, Carson G, Fukushima H, O'Brien J (1992). Structure and sequence of the human alpha-L-fucosidase gene and pseudogene. Genomics.

[18] Klontz EH, Li C, Kihn K, Fields JK, Beckett D, Snyder GA (2020). Structure and dynamics of an alpha-fucosidase reveal a mechanism for highly efficient IgG transfucosylation. Nat Commun.

[19] Johnson S (1991). Mammalian alpha-L-fucosidases. Comparative biochemistry and physiology.

[20] Hopfer R, Johnson S, Masserini M, Giuliani A, Alhadeff J (1990). Hydrolysis of fucosyl-GM1 ganglioside by purified pellet-associated human brain and human liver alpha-L-fucosidases without activator proteins or detergents. The Biochemical journal.

[21] Cordero O, Merino A, Páez de la Cadena M, Bugía B, Nogueira M, Viñuela J (2001). Cell surface human alpha-L-fucosidase. European journal of biochemistry.

[22] Becerra J, Rodríguez-Díaz J, Gozalbo-Rovira R, Palomino-Schätzlein M, Zúñiga M, Monedero V (2020). NUnique Microbial Catabolic Pathway for the Human Core-Glycan Constituent Fucosyl-α-1,6-Acetylglucosamine-Asparagine. mBio.

[23] Ali S, Jenkins Y, Kirkley M, Dagkalis A, Manivannan A, Crane IJ (2008). Leukocyte extravasation: an immunoregulatory role for alpha-L-fucosidase?. J Immunol.

[24] Phopin K, Nimlamool W, Lowe-Krentz L, Douglass E, Taroni J, Bean B (2013). Roles of mouse sperm-associated alpha-L-fucosidases in fertilization. Molecular reproduction and development.

[25] Liu T, Ho C, Huang H, Chang S, Popat S, Wang Y (2009). Role for alpha-L-fucosidase in the control of Helicobacter pylori-infected gastric cancer cells. Proceedings of the National Academy of Sciences of the United States of America.

[26] Xu L, Li Z, Song S, Chen Q, Mo L, Wang C (2020). Downregulation of alpha-l-fucosidase 1 suppresses glioma progression by enhancing autophagy and inhibiting macrophage infiltration. Cancer Sci.

[27] Tsuchida N, Ikeda M, Ιshino Υ, Grieco M, Vecchio G (2017). FUCA1 is induced by wild-type p53 and expressed at different levels in thyroid cancers depending on p53 status. International journal of oncology.

[28] Otero-Estevez O, Martinez-Fernandez M, Vazquez-Iglesias L, Paez de la Cadena M, Rodriguez-Berrocal FJ, Martinez-Zorzano VS (2013). Decreased expression of alpha-L-fucosidase gene FUCA1 in human colorectal tumors. Int J Mol Sci.

[29] Milde-Langosch K, Karn T, Schmidt M, zu Eulenburg C, Oliveira-Ferrer L, Wirtz RM (2014). Prognostic relevance of glycosylation-associated genes in breast cancer. Breast Cancer Res Treat.

[30] Vajaria BN, Patel PS (2017). Glycosylation: a hallmark of cancer?. Glycoconj J.

[31] Ezawa I, Sawai Y, Kawase T, Okabe A, Tsutsumi S, Ichikawa H (2016). Novel p53 target gene FUCA1 encodes a fucosidase and regulates growth and survival of cancer cells. Cancer Sci.

[32] Wang J, Ambros R, Weber P, Rosano T (1995). Fucosyltransferase and alpha-L-fucosidase activities and fucose levels in normal and malignant endometrial tissue. Cancer research.

[33] Gil-Martín E, Gil-Seijo S, Nieto-Novoa C, Fernández-Briera A (1996). Elevation of acid glycosidase activities in thyroid and gastric tumors. The international journal of biochemistry & cell biology.

[34] Vesce F, Biondi C (1983). alpha-L-fucosidase activity in endometrial, cervical and ovarian cancer. European journal of gynaecological oncology.

[35] Giardina M, Matarazzo M, Morante R, Lucariello A, Varriale A, Guardasole V (1998). Serum alpha-L-fucosidase activity and early detection of hepatocellular carcinoma: a prospective study of patients with cirrhosis. Cancer.

[36] Shah M, Telang S, Raval G, Shah P, Patel P (2008). Serum fucosylation changes in oral cancer and oral precancerous conditions: alpha-L-fucosidase as a marker. Cancer.

[37] Cheng T, Tu S, Chen L, Chen M, Chen W, Lin Y (2015). Down-regulation of α-L-fucosidase 1 expression confers inferior survival for triple-negative breast cancer patients by modulating the glycosylation status of the tumor cell surface. Oncotarget.

[38] Zhong A, Chen T, Xing Y, Pan X, Shi M (2021). FUCA2 Is a Prognostic Biomarker and Correlated With an Immunosuppressive Microenvironment in Pan-Cancer. Frontiers in immunology.

[39] Vogelstein B, Lane D, Levine AJ (2000). Surfing the p53 network. Nature.

[40] Lowe SW, Ruley HE, Jacks T, Housman DE (1993). p53-dependent apoptosis modulates the cytotoxicity of anticancer agents. Cell.

[41] Baudot AD, Crighton D, O'Prey J, Somers J, Sierra Gonzalez P, Ryan KM (2016). p53 directly regulates the glycosidase FUCA1 to promote chemotherapy-induced cell death. Cell Cycle.

[42] Kovacs E, Zorn J, Huang Y, Barros T, Kuriyan JJArob A structural perspective on the regulation of the epidermal growth factor receptor. 2015; 84: 739-64.

[43] Leahy DJAipc Structure and function of the epidermal growth factor (EGF/ErbB) family of receptors. 2004; 68: 1-27.

[44] Lemmon M, Schlessinger J, Ferguson KJCSHpib The EGFR family: not so prototypical receptor tyrosine kinases. 2014; 6: a020768.

[45] Zhen Y, Caprioli R, Staros JJB Characterization of glycosylation sites of the epidermal growth factor receptor. 2003; 42: 5478-92.

[46] Li W, Nakagawa T, Koyama N, Wang X, Jin J, Mizuno-Horikawa Y Down-regulation of trypsinogen expression is associated with growth retardation in alpha1,6-fucosyltransferase-deficient mice: attenuation of proteinase-activated receptor 2 activity. 2006; 16: 1007-19.

[47] Liu YC, Yen HY, Chen CY, Chen CH, Cheng PF, Juan YH (2011). Sialylation and fucosylation of epidermal growth factor receptor suppress its dimerization and activation in lung cancer cells. Proc Natl Acad Sci U S A.

[48] Thiery J, Acloque H, Huang R, Nieto MJC Epithelial-mesenchymal transitions in development and disease. 2009; 139: 871-90.

[49] Guo J, Li X, Tan Z, Lu W, Yang G, Guan F (2014). Alteration of N-glycans and expression of their related glycogenes in the epithelial-mesenchymal transition of HCV29 bladder epithelial cells. Molecules.

[50] Mizejewski GJPotSfEB, Biology MSfE, Medicine Role of integrins in cancer: survey of expression patterns. 1999; 222: 124-38.

[51] Gu J, Sumida Y, Sanzen N, Sekiguchi K (2001). Laminin-10/11 and fibronectin differentially regulate integrin-dependent Rho and Rac activation via p130(Cas)-CrkII-DOCK180 pathway. The Journal of biological chemistry.

[52] Janik M, Lityńska A, Vereecken PJBeba Cell migration-the role of integrin glycosylation. 2010; 1800: 545-55.

[53] Kawano T, Takasaki S, Tao TW, Kobata A (1993). Altered glycosylation of beta 1 integrins associated with reduced adhesiveness to fibronectin and laminin. International journal of cancer.

[54] Zhao Y, Itoh S, Wang X, Isaji T, Miyoshi E, Kariya Y Deletion of core fucosylation on alpha3beta1 integrin down-regulates its functions. 2006; 281: 38343-50.

[55] Liu D, Gao Z, Yue L (2019). Fucosyltransferase 8 deficiency suppresses breast cancer cell migration by interference of the FAK/integrin pathway. Cancer biomarkers: section A of Disease markers.

[56] Lu Y, Chen C, Chu C, Lu J, Wang B, Chen C Calreticulin activates β1 integrin via fucosylation by fucosyltransferase 1 in J82 human bladder cancer cells. 2014; 460: 69-78.

[57] Yuan K, Kucik D, Singh R, Listinsky C, Listinsky J, Siegal G (2008). Alterations in human breast cancer adhesion-motility in response to changes in cell surface glycoproteins displaying alpha-L-fucose moieties. International journal of oncology.

[58] Kusuma N, Anderson R, Pouliot NJC, metastasis e Laminin α5-derived peptides modulate the properties of metastatic breast tumour cells. 2011; 28: 909-21.

[59] Ruffell B, Coussens L (2015). Macrophages and therapeutic resistance in cancer. Cancer cell.

[60] Wang Y, Yan K, Lin J, Li J, Bi J (2021). Macrophage M2 Co-expression Factors Correlate With the Immune Microenvironment and Predict Outcome of Renal Clear Cell Carcinoma. Frontiers in genetics.

[61] Cameron D (1985). Specificity of macrophage-mediated cytotoxicity: role of target and effector cell fucose. Immunology letters.

[62] Donson J, Mandy K, Feng Z, Mandy S, Brown E, Godfrey H (1991). Role of monocyte fucose-receptors in T-cell fibronectin activity. Immunology.

[63] Rosati E, Mencarelli S, Magini A, Sabatini R, Tassi C, Orlacchio A (2007). Enhancement of lysosomal glycohydrolase activity in human primary B lymphocytes during spontaneous apoptosis. International journal of immunopathology and pharmacology.

[64] Crockard A, Bridges J, Lewis M (1980). A comparison of alpha-L-fucosidase activity in normal and chronic-lymphocytic-leukaemia lymphocytes. Biochemical Society transactions.

[65] Duan Q, Zhang H, Zheng J, Zhang L (2020). Turning Cold into Hot: Firing up the Tumor Microenvironment. Trends in cancer.

[66] Keeley T, Lin S, Lester D, Lau E, Yang S (2018). The fucose salvage pathway inhibits invadopodia formation and extracellular matrix degradation in melanoma cells. PloS one.

[67] Yuan K, Listinsky CM, Singh RK, Listinsky JJ, Siegal GP (2008). Cell surface associated alpha-L-fucose moieties modulate human breast cancer neoplastic progression. Pathol Oncol Res.

[68] Shuang Z, Mao Y, Lin G, Wang J, Huang X, Chen J (2018). Alpha-L-Fucosidase Serves as a Prognostic Indicator for Intrahepatic Cholangiocarcinoma and Inhibits Its Invasion Capacity. BioMed research international.

[69] Xing H, Qiu H, Ding X, Han J, Li Z, Wu H (2019). Clinical performance of α-L-fucosidase for early detection of hepatocellular carcinoma. Biomarkers in medicine.

[70] Waidely E, Al-Youbi A, Bashammakh A, El-Shahawi M, Leblanc R (2017). Alpha-l-Fucosidase Immunoassay for Early Detection of Hepatocellular Carcinoma. Analytical chemistry.

[71] Barlow J, DiCioccio R, Dillard P, Blumenson L, Matta K (1981). Frequency of an allele for low activity of alpha-L-fucosidase in sera: possible increase in epithelial ovarian cancer patients. Journal of the National Cancer Institute.

[72] Abdel-Aleem H, Ahmed A, Sabra A, Zakhari M, Soliman M, Hamed H (1996). Serum alpha L-fucosidase enzyme activity in ovarian and other female genital tract tumors. International journal of gynaecology and obstetrics: the official organ of the International Federation of Gynaecology and Obstetrics.

[73] Wang K, Guo W, Li N, Shi J, Zhang C, Lau WY (2014). Alpha-1-fucosidase as a prognostic indicator for hepatocellular carcinoma following hepatectomy: a large-scale, long-term study. Br J Cancer.

[74] Yu X, Zhang R, Yang T, Zhang M, Xi K, Lin Y (2019). Alpha-l-fucosidase: a novel serum biomarker to predict prognosis in early stage esophageal squamous cell carcinoma. J Thorac Dis.

[75] Ayude D, Páez de la Cadena M, Cordero O, Nogueira M, Ayude J, Fernández-Briera A (2003). Clinical interest of the combined use of serum CD26 and alpha-L-fucosidase in the early diagnosis of colorectal cancer. Disease markers.

[76] Zhang S, Lin B, Li B (2015). Evaluation of the diagnostic value of alpha-l-fucosidase, alpha-fetoprotein and thymidine kinase 1 with ROC and logistic regression for hepatocellular carcinoma. FEBS open bio.

[77] Chen D, Xing N, Cui Z, Zhang C, Zhang Z, Li D (2020). The Value of Preoperative Alpha-L-Fucosidase Levels in Evaluation of Malignancy and Differential Diagnosis of Urothelial Neoplasms. Journal of oncology.

[78] Wang YN, Lee HH, Hsu JL, Yu D, Hung MC (2020). The impact of PD-L1 N-linked glycosylation on cancer therapy and clinical diagnosis. Journal of biomedical science.

[79] Mondal N, Silva M, Castano AP, Maus MV, Sackstein R (2019). Glycoengineering of chimeric antigen receptor (CAR) T-cells to enforce E-selectin binding. The Journal of biological chemistry.

[80] Cai L, Gu Z, Zhong J, Wen D, Chen G, He L (2018). Advances in glycosylation-mediated cancer-targeted drug delivery. Drug discovery today.

[81] Hottin A, Wright DW, Steenackers A, Delannoy P, Dubar F, Biot C (2013). α-L-fucosidase inhibition by pyrrolidine-ferrocene hybrids: rationalization of ligand-binding properties by structural studies. Chemistry (Weinheim an der Bergstrasse, Germany).

[82] Jiang J, Kallemeijn W, Wright D, van den Nieuwendijk A, Rohde V, Folch E (2015). In vitro and comparative and competitive activity-based protein profiling of GH29 α-l-fucosidases. Chemical science.

[83] Dubernet M, Defoin A, Tarnus C (2006). Asymmetric synthesis of the L-fuco-nojirimycin, a nanomolar alpha-L-fucosidase inhibitor. Bioorganic & medicinal chemistry letters.

[84] Zhou J, Negi A, Mirallai S, Warta R, Herold-Mende C, Carty M (2019). N-Alkyl-1,5-dideoxy-1,5-imino-l-fucitols as fucosidase inhibitors: Synthesis, molecular modelling and activity against cancer cell lines. Bioorganic chemistry.

[85] Winchester B, Barker C, Baines S, Jacob G, Namgoong S, Fleet G (1990). Inhibition of alpha-L-fucosidase by derivatives of deoxyfuconojirimycin and deoxymannojirimycin. The Biochemical journal.

[86] Pinsky L, Callahan J, Wolfe LJL Fucosidosis?. 1968; 2: 1080.

[87] Asano N, Yasuda K, Kizu H, Kato A, Fan J, Nash R (2001). Novel alpha-L-fucosidase inhibitors from the bark of Angylocalyx pynaertii (Leguminosae). European journal of biochemistry.

[88] Kato A, Okaki T, Ifuku S, Sato K, Hirokami Y, Iwaki R (2013). Synthesis and biological evaluation of N-(2-fluorophenyl)-2β-deoxyfuconojirimycin acetamide as a potent inhibitor for α-l-fucosidases. Bioorganic & medicinal chemistry.

[89] Mondon M, Lecornué F, Guillard J, Nakagawa S, Kato A, Blériot Y (2013). Skeletal rearrangement of seven-membered iminosugars: synthesis of (-)-adenophorine, (-)-1-epi-adenophorine and derivatives and evaluation as glycosidase inhibitors. Bioorganic & medicinal chemistry.

[90] Ak A, Prudent S, LeNouën D, Defoin A, Tarnus C (2010). Synthesis of all-cis 2,5-imino-2,5-dideoxy-fucitol and its evaluation as a potent fucosidase and galactosidase inhibitor. Bioorganic & medicinal chemistry letters.

[91] Stocker B, Jongkees S, Win-Mason A, Dangerfield E, Withers S, Timmer M (2013). The 'mirror-image' postulate as a guide to the selection and evaluation of pyrrolidines as α-l-fucosidase inhibitors. Carbohydrate research.

[92] Hottin A, Scandolera A, Duca L, Wright D, Davies G, Behr J (2016). A second-generation ferrocene-iminosugar hybrid with improved fucosidase binding properties. Bioorganic & medicinal chemistry letters.

[93] Okada M, Chikuma S, Kondo T, Hibino S, Machiyama H, Yokosuka T (2017). Blockage of Core Fucosylation Reduces Cell-Surface Expression of PD-1 and Promotes Anti-tumor Immune Responses of T Cells. Cell reports.

[94] Takeshita H, Yasuda T, Nadano D, Iida R, Nakanaga M, Tenjo E (1994). Genetically polymorphic alpha-L-fucosidase (FUCA1) isozymes detected in blood plasma. Human genetics.

[95] Krause A, Combaret V, Iacono I, Lacroix B, Compagnon C, Bergeron C (2005). Genome-wide analysis of gene expression in neuroblastomas detected by mass screening. Cancer letters.

[96] Ohira M, Morohashi A, Inuzuka H, Shishikura T, Kawamoto T, Kageyama H (2003). Expression profiling and characterization of 4200 genes cloned from primary neuroblastomas: identification of 305 genes differentially expressed between favorable and unfavorable subsets. Oncogene.

[97] Reglero A, Carretero M, Cabezas J (1980). Increased serum alpha-L-fucosidase and beta-N-acetylglucosaminidase activities in diabetic, cirrhotic and gastric cancer patients. Clinica chimica acta; international journal of clinical chemistry.

[98] Yu XY, Lin SC, Zhang MQ, Guo XT, Ma K, Wang LX (2022). Association and prognostic significance of alpha-L-fucosidase-1 and matrix metalloproteinase 9 expression in esophageal squamous cell carcinoma. World journal of gastrointestinal oncology.

[99] Hutchinson W, Johnson P, Du M, Williams R (1991). Serum and tissue alpha-L-fucosidase activity in the pre-clinical and clinical stages of hepatocellular carcinoma. Clinical science (London, England: 1979).

[100] Vajaria BN, Patel KR, Begum R, Shah FD, Patel JB, Shukla SN (2013). Evaluation of serum and salivary total sialic acid and α-l-fucosidase in patients with oral precancerous conditions and oral cancer. Oral surgery, oral medicine, oral pathology and oral radiology.

[101] Vajaria BN, Patel KA, Patel PS (2018). Role of aberrant glycosylation enzymes in oral cancer progression. Journal of carcinogenesis.

[102] Amos C, Struewing J (1993). Genetic epidemiology of epithelial ovarian cancer. Cancer.

[103] Liu J, Lin B, Hao Y, Qi Y, Zhu L, Li F (2009). Lewis y antigen promotes the proliferation of ovarian carcinoma-derived RMG-I cells through the PI3K/Akt signaling pathway. Journal of experimental & clinical cancer research: CR.

